# Impact of Malakit intervention on perceptions, knowledge, attitudes, and practices related to malaria among workers in clandestine gold mines in French Guiana: results of multicentric cross-sectional surveys over time

**DOI:** 10.1186/s12936-022-04391-4

**Published:** 2022-12-28

**Authors:** Cécile Longchamps, Muriel Suzanne Galindo, Yann Lambert, Alice Sanna, Louise Mutricy, Laure Garancher, Antoine Adenis, Mathieu Nacher, Martha Suarez-Mutis, Hedley Cairo, Helen Hiwat, Stephen Vreden, Maylis Douine

**Affiliations:** 1 Centre d’Investigation Clinique Antilles-Guyane, Inserm 1424, Centre Hospitalier de Cayenne Andrée Rosemon, Cayenne, French Guiana France; 2 TheInkLink Association, Paris, France; 3 TBIP, Université de Guyane, Cayenne, French Guiana France; 4 Laboratory of Parasitic Diseases, Institute Oswaldo Cruz, Fiocruz, Rio de Janeiro, Brazil; 5 National Malaria Programme of Suriname, Paramaribo, Suriname; 6 Foundation for the Advancement of Scientific Research in Suriname, Paramaribo, Suriname

**Keywords:** Mobile population, Hard-to-reach population, Malaria elimination, Knowledge, Attitudes, Practices, Propensity score analysis, Amazon forest

## Abstract

**Background:**

Clandestine gold miners remain key hosts for malaria in French Guiana (FG) and contribute to imported malaria cases in Suriname and Brazil. The Malakit intervention, implemented in FG borders with Suriname and Brazil, provided gold miners with training on malaria and kits for self-diagnosis and self-treatment. Having shown a likely impact on malaria transmission, Suriname has now implemented it in routine care for cross-border moving populations. However, a decrease in malaria transmission is frequently associated with a decrease in risk perception, knowledge, and good practices regarding malaria. This study aims to describe the evolution of the perceptions, knowledge, attitudes, and practices (KAP) related to malaria among clandestine gold miners between 2015 and 2019, and to estimate the impact of Malakit on the FG/Suriname border.

**Methods:**

The primary outcome was the overall KAP score over time and among participants and not participants in the Malakit intervention. A propensity score matching analysis and an inverse probability of treatment weighing analysis were used to estimate the Average Treatment effect on the Treated and the Average Treatment Effect of Malakit, respectively.

**Results:**

Perception and knowledge scores were significantly lower in 2019 compared to 2015 (− 0.27 and − 0.23 points, respectively, p < 0.001) while attitude and practice scores were higher (+ 0.16 and + 0.47 points, respectively, p < 0.001). The overall KAP score was significantly higher among participants in Malakit with both propensity score matching (+ 0.72 points, 95%IC [0.29; 1.15]) and inverse probability of treatment weighting analysis (+ 0.70 points, 95%IC [0.34; 1.05]).

**Conclusion:**

A decrease in perception and knowledge about malaria but an improvement of attitudes and practices as the incidence of malaria decreased are observed. The Malakit intervention seems to have a significant positive impact on the overall KAP related to malaria. The integration of this strategy into malaria control programmes could help to improve the KAP, even in areas where malaria is nearly eliminated, through optimal training and health empowerment.

*Trial registration* ClinicalTrials.gov registration number: NCT03695770.

**Supplementary Information:**

The online version contains supplementary material available at 10.1186/s12936-022-04391-4.

## Background

In 2020, the worldwide number of malaria cases was estimated at 241 million, causing approximately 627,000 deaths. While malaria strategies led to favourable trends between 2000 and 2015, the main indicators of morbidity and mortality have remained broadly stable since 2015, with local variations [1]. One of the factors associated with the continuation or resurgence of malaria transmission is human mobility, particularly in cross-border contexts and with the progressive anthropization of natural environments [2, 3]. Gold mining areas in the Guiana Shield, a region encompassing French Guiana (FG), Suriname, Guyana and states of Venezuela, Colombia, and Brazil, are often characterized by a high malaria endemicity. Miners can sustain malaria reintroduction in low burden areas because of their frequent cross-border mobility [4].

FG shares land borders with Brazil (Amapá state) and Suriname. Malaria incidence has decreased in all three territories in the last 15 years [5–7], but FG is still the only territory in the European Union where autochthonous transmission of malaria is ongoing. The transmission is mainly concentrated in gold mining areas which are usually located more than a day away from the nearest healthcare centre [8–10]. Moreover, the delivery of malaria diagnosis and treatment by community health workers at gold mining sites—like in Suriname—are not permitted under French regulations. Therefore, clandestine gold miners in FG are not reached by the health system even if care in health centres is free of charge. In 2016, the World Health Organization (WHO) included Suriname among the 21 countries that could eliminate malaria by 2020 [11]. However, malaria elimination in Suriname has been challenged by cross-border cases imported from clandestine gold mining areas in FG [8].

An innovative case management approach, called Malakit, was developed and implemented between 2018 and 2020 resulting from collaboration between Cayenne Hospital (FG), Suriname, and Brazil, to address malaria diagnosis and effective treatment of clandestine gold miners in FG. Ultimately, these efforts aimed to reduce the risk of emergence of resistant *Plasmodium falciparum* posed by the use of inappropriate treatment by the target population [12–16]. This project consisted in distributing kits containing malaria self-tests and Artemisinin-based combination therapy (ACT) to be used in the gold mining areas in FG, as well as training on malaria and how to correctly self-diagnose and self-treat. From April 2018 to March 2020, 4,766 kits were distributed to 3733 participants. The estimated coverage of the target population was estimated at 29.7%.

The evaluation of the Malakit intervention showed a significant improvement of self-care behaviour: the proportion of gold miners reporting proper treatment with an ACT after a malaria diagnosis in the event of malaria symptoms significantly increased (OR = 1.8, 95% CI [1.1; 3.0]) [14]. The project has presumably contributed to the reduction of malaria transmission in the region: the incidence and prevalence of malaria decreased significantly in FG and the surrounding territories (Suriname, Amapá), in clandestine gold mines as well as in local inhabitants and so did the number of imported malaria cases from FG to Suriname or Brazil [17, 18]. An inversion of the proportion of *P. falciparum* and *Plasmodium vivax* with more than 85% of *P. vivax* was also found in the gold mining population. This predominance of *P. vivax* is typically found in areas that have reached the control phase of malaria and are entering the elimination phase [19]. These results suggest that the elimination of malaria may be feasible in FG by 2025. Another typical consequence of the decrease in malaria transmission in pre-elimination contexts is a decrease in malaria risk perception, knowledge about the disease, practice of the preventive measures [20–23].

It is, therefore, important to maintain efforts on prevention and access to diagnosis and treatment in the elimination phase to achieve the target set by WHO’s Sustainable Development Goals: ‘by 2030, end the epidemics of AIDS, tuberculosis, malaria and neglected tropical diseases and combat hepatitis, water-borne diseases and other communicable diseases’ [24–27].

Given the positive results of the Malakit project, the Malaria Programme in Suriname has implemented the Malakit intervention in routine care for cross-border moving populations. Moreover, a new project is being developed, still in collaboration with Suriname and Brazil, to implement and evaluate a complementary intervention to Malakit that would target *P. vivax* by treating probable hypnozoites carriers (based on epidemiological data and *P. vivax* serology) by 8-amnioquinoleine.

In this context, to better understand and adapt the information provided to gold miners to achieve malaria elimination in FG and Suriname, this study aims to evaluate the evolution of perceptions, knowledge, attitudes, and practices (KAP) related to malaria before and after the Malakit intervention (2015 versus 2019) and for participants in the intervention vs. non-participants.

The objectives were:

- to describe the evolution of the KAP among clandestine gold miners between 2015 and 2019
- to estimate the Average Treatment effect on the Treated (ATT), i.e. the effect of Malakit on the KAP among gold miners included in the intervention between 2018 and 2019
- to estimate the Average Treatment Effect (ATE) of Malakit on the KAP related to malaria, i.e. the potential effect of Malakit on the KAP if all the gold miners passing through resting sites on the FG-Suriname border could be included and receive Malakit training.

## Methods

### Malakit project

Malakit is an international and multicentric interventional project with a quasi-experimental design, based on a single group intervention and an independent pre- and post-intervention evaluation survey including participants regardless of their participation or non-participation in the Malakit intervention. The project was coordinated by Cayenne Hospital in FG with one principal coordinating investigator, one intervention coordinator, one evaluation coordinator and one doctoral student. There were two principal investigators—one from Suriname and one from Brazil, and two local field supervisors—one on each FG border. The methodology has been previously published [13, 15], and further details are available at www.malakit-project.org.

#### Malakit intervention strategy

The strategy of the intervention is to deliver to persons working on clandestine gold mining sites in FG, a kit (malakit) to enable them to self-diagnose and self-treat when they are in the forest on the gold mining sites in FG. The kits are delivered on resting sites, specific neighbourhoods in border towns or small informal settlements located on border rivers where gold miners come to rest, buy supplies or sell gold. Inclusion criteria were being aged 15 years and above and working on clandestine gold mining sites in FG.

This ‘malakit’ includes three all-species rapid diagnostic tests (RDT), a full artemisinin-based combination therapy (ACT) course, which is the standard treatment, a single dose of primaquine for early clearance of *P. falciparum* gametocytes, and paracetamol. Before receiving a malakit, participants to the intervention received a training on malaria and on the correct use of the kit. As shown in Table 1, the training included information about malaria transmission and symptoms, its diagnosis, its treatment with importance of adherence and what to do in case of treatment failure, and its prevention (Additional file 1) [12, 15].

**Table 1:** Content of the participants’ malaria training

Malaria training themes	Key points
Transmission	---Vector, mosquito bite ---Parasites, the difference between *P. vivax* and *P. falciparum*
Symptoms	---Regular malaria symptoms ---Severe symptoms
Diagnosis	---Importance of knowing the cause of the infection before taking a treatment ---How to perform and interpret the self-test ---What to do in case of negative test
Treatment	---Treatment course in case of positive test ---Contraindications ---Adverse events ---Why complete all the treatment (efficacy, resistance) ---What to do in case of severe, persistent or relapse of symptoms
Prevention	---Reminder of the main prevention measures (mosquito nets, repellent on the skin…)

Nine community health workers called “facilitators” were in charge of training and delivering the kits. They belonged to the gold miner community and were fluent or native Portuguese speakers.

The training tools were developed through a participatory approach [15] and included posters, illustrated instructions on the plastic pouch of the malakit, videos and a smartphone application containing these videos (Additional file 2). The illustrated pouch of the malakit as well as the application allowed participants to leave with easily usable and understandable information material on malaria and its management for themselves as well as for the other gold miners in a peer-to-peer education approach.

Implementation evaluation showed that the training was highly interactive [12, 16]. The facilitators adapted the duration of the explanations, the focus on specific messages, and the vocabulary to the participant characteristics (comorbidities, literacy), their understanding during the training and their availability in time [12]. Special attention was given by the facilitators on the comprehension of the correct use of the malakit i.e. self-diagnosis and self-treatment. Information on the transmission by mosquito bite and the ways to prevent it (at least the use of mosquito nets) were systematically mentioned. An insecticide-impregnated bed net was also distributed to the participants.

**Fig. 1:**
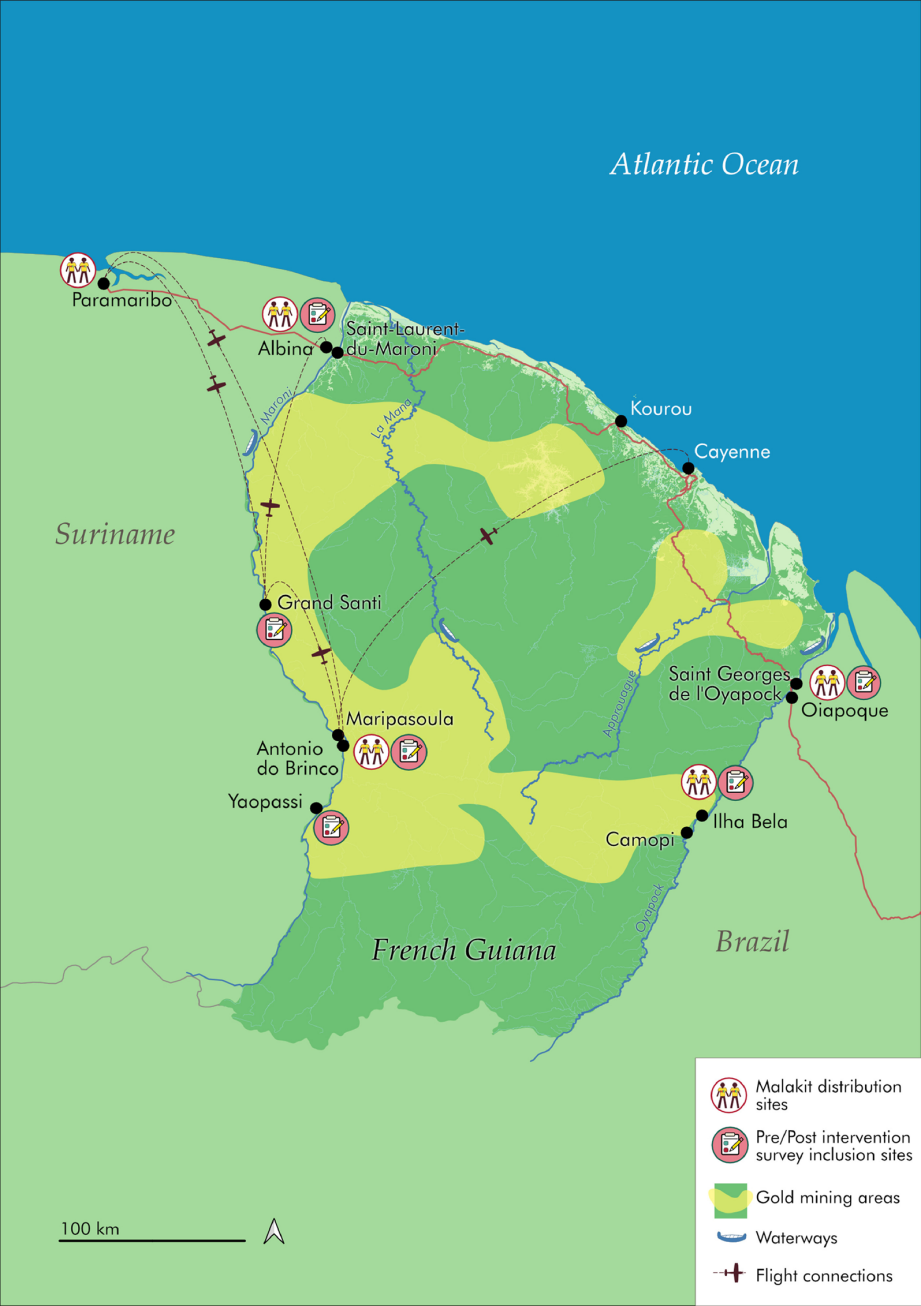
Inclusion sites for pre/post-intervention surveys and Malakit intervention. Source: Douine et al., The Lancet Regional Health – Americas, 2021 [14]

Inclusions in the Malakit intervention and follow-up visits were performed between April 2018 and March 2020, with a progressive roll-out in the five inclusion sites located at resting sites for gold miners: two on the FG-Brazil border, two on the FG-Suriname border and one site in Paramaribo, the capital of Suriname (Fig. 1).

#### Pre-post evaluation surveys

Two cross-sectional surveys, before and after the Malakit intervention assessing KAP and malaria prevalence, were conducted. The gold miners were recruited by snowball sampling method at the resting areas where gold miners entered FG from Brazil (two inclusion sites) and Suriname (four inclusion sites). Inclusion criteria were being 18 years old or more working or accompanying someone on a clandestine gold mining site in FG, being out of the forest for less than 7 days, giving written consent.

In this study, the data collected along the FG-Suriname border are analaysed to continuously improve the Malakit intervention now implemented by the Surinamese Malaria Programme. The pre-intervention survey took place from January to June 2015, and the post-intervention survey from October to December 2019, in four sites along the Maroni River at the FG-Suriname border (Fig. 1).

### Data collection

Once participants’ consent was obtained, questionnaires were administered by a mediator, different from the intervention’s facilitators (Additional file 3). Data were collected on paper or with tablets that transferred anonymized data to a secure online database. The sociodemographic profile of the gold miners was assessed by collecting data on gender, age, level of education, country of birth, proficiency in Portuguese, medical coverage in France. Questions regarding the gold mining activity included the time since their first working experience in gold mining and the main activities. Finally, data on the history of malaria, the last episode of malaria, the risk perception and knowledge related to malaria, the attitudes towards malaria treatment and the practice of preventive measures were collected. Two questions about the attitude towards testing and treatment were only asked in the post-intervention survey. The answer modalities of the question about malaria transmission were different in pre and post-intervention survey since a difference was made between “mosquito bite” and “mosquito” in the post-intervention survey while the answer “mosquito” included “mosquito bite” in the pre-intervention survey.

### Outcomes

#### Primary outcome

The main outcome of this study was the 12 points KAP score after the intervention (Table 2).

**Table 2:** Scores calculation

Scores and details of the scores	Before/after	Included/ not included in Malakit
**Perception score**	**3 points**	**3 points**
Malaria is a major health problem	1	1
Malaria is deadly	1	1
Malaria cannot be cured without treatment	1	1
**Knowledge score**	**2 points**	**3 points**
Transmission by mosquitoes	1	/
Transmission by mosquito bites/mosquitoes	/	2
≥ 3 symptoms of malaria known	1	1
**Attitude score**	**2 points**	**4 points**
Diagnostic test before treatment	/	1
No treatment if the test is negative	1	1
No treatment interruption until the end	1	1
Health professionals' treatments are better than those on the black market	/	1
**Practice score**	**2 points**	**2 points**
Frequent protection against mosquitos	1	1
Last night on gold mining site was under a mosquito net	1	1
**KAP scores**	**9 points**	**12 points**

Primary and secondary outcomes are shown in bold

#### Secondary outcome

The secondary outcomes of this study were the 9-point KAP score, the 3-point perception score, the 2-point and 3-point knowledge scores, the 2-point and 4-point attitude scores and the 2-point practice score (Table 2).

### Analysis

#### Score calculation

The different scores were calculated as shown in Table 2 (Additional file 4).

#### Statistical analysis

Frequencies and proportions were calculated for qualitative variables, and median, interquartile range and standard deviation for quantitative variables. Bivariate analyses were conducted between pre-intervention and post-intervention groups then between the participants included and not included in Malakit in the post-intervention group, using Student’s test for quantitative variables, and Chi2 or exact Fisher tests for qualitative variables.

The literature shows strong evidence of the utility and efficacy of using propensity score analysis to estimate the causal effect of public health intervention in the context of quasi-experimental studies to reduce selection and causality bias due to lack of randomization [28, 29]. The propensity score is the probability of treatment assignment conditional on the measured baseline covariates so that conditional on the true propensity score, treatment status is independent of the measured baseline covariates [30]. Among the different propensity score (PS) analysis methods, Propensity Score Matching (PSM) and Inverse Probability of Treatment Weighting (IPTW) are those recommended to estimate the Average Treatment effect on the Treated (ATT) and the Average Treatment Effect (ATE), respectively [31]. Thus, a one-to-one nearest-neighbour PSM analysis was performed on the post-intervention group to estimate the effect of the Malakit intervention on gold miners who get the intervention and an IPTW analysis to estimate the effect of the Malakit intervention on the overall target population (i.e. all the gold miners frequenting the Malakit distribution sites in Suriname). The variables associated with both the inclusion in Malakit and the KAP score or the variables associated only with the KAP score were included in the logistic regression model to estimate the propensity score [31–33]: sex, age, level of education, proficiency in Portuguese, time worked in gold mining, history of malaria, French health insurance coverage. Participants with missing data for these variables or the variables used to calculate the scores were excluded from the propensity score analysis (N = 26/380). The covariates balance between participants included and not included in Malakit before and after the propensity score estimation was assessed using the absolute value of the standardized mean difference (SMD) for each covariate. A |SMD|< 0.1 was considered an adequate balanced and a |SMD|> 0.2 a serious imbalance [34–36]. Outcome analysis was performed using a linear regression model with a paired t-test for the PSM analysis, and a linear marginal structural model with a t-test for the IPTW analysis. A 5% significance threshold was fixed. Sensitivity analysis to determine the robustness of the results to hidden biases was performed using the Rosenbaum Sensitivity Test for Wilcoxon Signed Rank P-Value for the PSM analysis [37] and optimal trimming for IPTW analysis [38] (Additional file 7).

The effect size of the Student test was calculated using Cohen’s d measure. A effect size inferior to 0.2 was considered negligible, minimal if it was between 0.2 and 0.5 and important if it was superior to 0.5 [39]. Statistical analysis was performed with RStudio© software version 1.4.1103. The PSM analysis was performed with the MatchIt [40] and Rbounds [41] packages and the IPTW analysis with the PSweight [42] and Survey [43] packages.

## Results

### Participation

421 participants were included in the pre-intervention survey in 2015 and 380 in the post-intervention survey in 2019. Among the 380 post-survey participants, 30.8% (N = 117) were included in Malakit.

### Population characteristics

Participants included in the pre- and post-survey were mainly male, aged between 30 and 45 years, born in Brazil, working in gold mining for about 10 years (Table 3).

**Table 3:** Pre- and post-intervention surveys’ population characteristics

		Pre-intervention survey (2015) (N = 421) N (%)	Post-intervention survey (2019) (N = 380) N (%)
Sex	Female	124 (29.5)	102 (26.8)
	Male	297 (70.6)	278 (73.2)
Age	Median (IQR)	37 (30–45)	39 (31–48)
	18–29	91 (21.6)	80 (21.1)
	30–44	214 (50.8)	168 (44.2)
	≥ 45	116 (27.6)	132 (34.7)
Country of birth	Brazil	395 (93.8)	363 (95.5)
	Other	26 (6.2)	17 (4.5)
Education level	None or primary	202 (48.0)	130 (34.3)
	Secondary or superior	219 (52.0)	249 (65.7)
Portuguese level	None or low	13 (4.0)	4 (1.1)
	Fluent	408 (96.9)	370 (98.9)
French medical coverage	Yes	13 (3.1)	10 (2.6)
	No	408 (96.9)	370 (97.4)
Time in gold mining	Median (IQR)	10 (5–15)	10 (4–18)
	≤ 10 years	249 (59.1)	205 (54.1)
	> 10 years	172 (40.9)	174 (45.9)
Main occupation	Gold miner	206 (48.9)	230 (60.5)
	Trader	92 (21.9)	59 (15.5)
	Cook/Housekeeper/Sex worker	65 (15.4)	55 (14.5)
	Quad or boat driver/Mechanic	48 (11.4)	23 (6.1)
	Other	10 (2.4)	13 (3.4)
History of malaria	Never or < 4 attacks > 4 attacks	136 (32.3) 285 (67.7)	184 (48.8) 193 (51.2)

### Evolution of KAP between 2015 and 2019

**Fig. 2:**
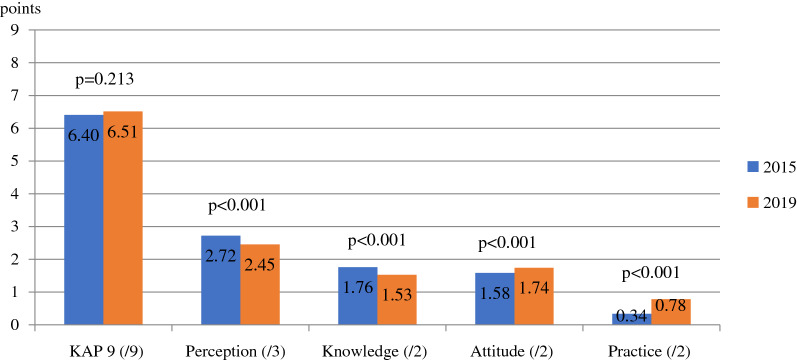
Evolution of the KAP scores before (N = 421) and after (N = 380) the intervention

Between 2015 and 2019, the bivariate analysis showed an overall stability of the KAP 9 score (+ 0.11 points, p = 0.213, Cohen’s d = 0.556) but significant decrease for each dimension of the KAP score (Fig. 2).

The perception of the risk related to malaria was lower in 2019 (− 0.27 points, p < 0.001, d = 0.292). While in 2015, 84.8% of participants considered malaria to be a major health problem, only 55.3% cited malaria as one of the top three health problems on the resting sites in 2019 (Additional file 5).

Similarly, knowledge about the transmission of the disease and its symptoms decreased over time (− 0.23 points, p < 0.001, d = 0.365). Indeed, the mosquito was identified as the cause of malaria by 90.7% of participants in 2015 by 80.8% in 2019, and 85.5% knew at least 3 symptoms in 2015 versus 72.1% in 2019. In contrast, the attitude towards diagnosis and compliance with treatment improved during this period (+ 0.16 points, p < 0.001, d = 0.151).

Finally, a better practice of malaria preventive measures (+ 0.47 points, p < 0.001, d = 0.298) is observed, mainly through the use of impregnated mosquito nets.

### Effect of Malakit on KAP

#### Bivariate analysis

**Fig. 3:**
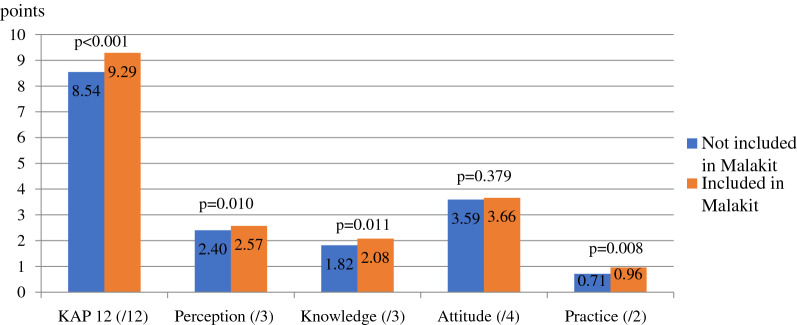
Bivariate analysis of KAP among Malakit participants (N = 107) and non-participants (N = 247)

As shown in Fig. 3, an overall significant improvement in the KAP 12 score for those who participated in Malakit in bivariate analyses (+ 0.75 points, p < 0.001, d = 0.479) is observed. This improvement concerns almost all the dimensions of the KAP score: + 0.17 points for the perception score (p = 0.010, d = 0.292), + 0.75 for the knowledge score (p = 0.011, d = 0.285), + 0.25 for the practice score (p = 0.008, d = 0.298) (Additional file 6). In bivariate analysis, attitudes towards diagnosis and treatment were not significantly better among Malakit participants compared to non-participants (+ 0.24 points, p = 0.379, d = 0.098).

#### Propensity score estimation and covariates balance

All the covariates assumed to be potential confounders were imbalanced between the participants and non-participants in Malakit intervention (|SMD|> 0.1) in the initial study population (Table 4).

**Table 4:** Covariates balance between participants and non-participants in Malakit before and after PS estimation

**Covariates**	Initial (N=354)				Propensity score matching (N=214)			Inverse probability of treatment weighting (N=354)		
	Not included in Malakit (N=247)	Included in Malakit (N=107)		|SMD|	Not included in Malakit (N=107)	Included in Malakit (N=107)	|SMD|	Not included in Malakit (N=247)	Included in Malakit (N=107)	|SMD|
Male (%)	78.1	67.3		0.244**	66.4	67.3	0.020	74.6	74.2	0.009
Age (years)	41.2	36.9		0.403**	36.6	36.9	0.035	39.9	40.0	0.012
None or primary education level (%)	39.7	25.2		0.311**	23.4	25.2	0.043	35.3	35.1	0.005
Not fluent in Portuguese (%)	1.2	0.9		0.027	1.9	0.9	0.097	1.1	1.2	0.006
No French health coverage (%)	98.0	96.3		0.102*	98.1	96.3	0.099	97.4	97.3	0.005
Time worked in gold mining (years)	13.7	10.7		0.292**	10.3	10.7	0.047	12.9	13.0	0.012
<4 malaria episodes experienced (%)	46.6	49.5		0.059	52.3	49.5	0.056	47.4	46.7	0.015

^*^|Standardized mean difference| between 0.1 and 0.2

^**^|SMD|> 0.2

As shown in Table 4, the sub-population after PSM on the nearest neighbour includes 214 participants: 107 included in Malakit, 107 not included in Malakit and all the covariates were balanced. After IPTW, the distribution of measured baseline covariates was similar between participants included and not included in Malakit (Additional file 7).

#### Average effect of Malakit on participants

As shown in Table 5, a significant positive overall ATT effect of Malakit is observed since the participants to the intervention had a mean KAP 12 score equal to 9.33, which is 0.72 points higher than non-participants (p = 0.001, d = 0.357). The difference was significant in knowledge (+ 0.34 points, p = 0.010, d = 0.287) and practice (+ 0.25 points, p = 0.025, d = 0.215) scores. No significant differences were observed in perception (p = 0.380, d = 0.084) and attitude (p = 0.439, d = 0.081) scores.

**Table 5:** Outcome analysis after PSM among participants and non-participants in Malakit

**Score**	Not included in Malakit (N=107)Mean (sd)	Included in Malakit (N=107)Mean (sd)	Coefficient [CI95%]	p-value
KAP 12 (/12)	8.61 (1.52)	9.33 (1.58)	0.72 [0.29; 1.15]	0.001*
Perception (/3)	2.50 (0.57)	2.56 (0.54)	0.07 [-0.08; 0.21]	0.380
Knowledge (/3)	1.75 (0.96)	2.08 (0.81)	0.34 [0.08; 0.59]	0.010*
Attitude (/4)	3.64 (0.60)	3.71 (0.58)	0.07 [-0.07; 0.22]	0.439
Practice (/2)	0.72 (0.83)	0.97 (0.84)	0.25 [0.03; 0.47]	0.025*

^*^p < 0.05 using paired t-test

#### Potential average effect of Malakit

As shown in Table 6, the mean of the KAP 12 score among potential participants to Malakit was 9.32, 0.70 higher than the non-participants mean (p < 0.001), showing a significant positive overall ATE effect of the intervention. When looking at the specific dimensions, the difference was significant on the knowledge (+ 0.021, p = 0.018) and the practice (+ 0.26, p = 0.012) scores.

**Table 6:** Outcomes analysis after IPTW among participants and non-participants in Malakit

**Score**	Not included in Malakit (N=107)Mean (sd)	Included in Malakit (N=107)Mean (sd)	Coefficient [CI95%]	p-value
KAP 12 (/12)	8.62 (1.52)	9.32 (1.53)	0.70 [0.34; 1.05]	<0.001*
Perception (/3)	2.41 (0.61)	2.52 (0.57)	0.10 [-0.04; 0.25]	0.161
Knowledge (/3)	1.87 (0.94)	2.11 (0.80)	0.24 [0.04; 0.43]	0.018*
Attitude (/4)	3.62 (0.64)	3.72 (0.55)	0.10 [-0.03; 0.23]	0.136
Practice (/2)	0.72 (0.83)	0.97 (0.83)	0.26 [0.06; 0.46]	0.012*

^*^p < 0.05 using t-test

## Discussion

This study shows a significant decrease on perception and knowledge towards malaria between 2015 and 2019 concomitant with the decrease in incidence while improved attitudes and practices. It also reveals that participation to the Malakit intervention was associated to improved knowledge and preventive practices related to malaria among gold miners. This information is crucial in the context of malaria elimination in Suriname and French Guiana to avoid malaria resurgence.

### Strengths and limitations

Some limitations should be noted: i) lower statistical power after matching; ii) desirability bias regarding attitudes towards diagnosis and treatment and preventive behaviour answers, reduced by the administration of the questionnaire by a mediator familiar with the gold miners’ community iii) scores not evaluated in the literature, based on previous studies in the study population [44].

These results appear to be robust given their consistency between the PSM and IPTW analysis and the results of the sensitivity analysis (Additional file 7). The consistency of these results also suggests that most confounding factors were accounted for when estimating the propensity scores. The use of propensity score analysis, a validated method to evaluate the impact of a public health intervention in a quasi-experimental setting, limits selection and confounding biases [28–30].

### Contrasting evolution of the KAP related to malaria over time

The evolution of gold miners KAP between 2015 and 2019 shows a significant decrease in the perception of malaria risk and knowledge about the transmission and symptoms of the disease). Another recent study found similar results among gold miners in the FG/Brazil area [45]. This phenomenon is frequently observed in regions achieving malaria control, due to the reduced contact of the population with the disease [20, 46].

In contrast with the literature, a parallel significant improvement of knowledge regarding diagnosis and treatment as well as in preventive behaviour, such as the use of bed nets is observed, even if the practice of preventive measures remains low (practice score mean in 2019: 0.78/2). This paradoxical evolution could be explained by the distribution of mosquito nets by the facilitators at the same time as the delivery of the malakits, as well as by the team's advocacy to ensure that the kits and the nets were not destroyed during military operations on the gold-mining sites.

Another hypothesis to explain the improvement of KAP as a result of Malakit intervention is the dissemination of the information delivered through the facilitators, mainly addressing the behaviour in case of symptoms both with the training and the kit delivery, by the participants to the non-participants [14].

### A positive impact of Malakit on gold miners’ KAP

Malakit seemed to have a significant impact on the overall knowledge, attitudes and practices related to malaria of gold miners. The participation in the intervention increased their KAP 12 score compared to non-participants between 2018 and 2019. Furthermore, if the routine implementation of Malakit by the Suriname Malaria Program allowed for the inclusion of all cross-border moving gold miners from FG to Suriname, the KAP 12 score mean would increase in the target population. In these results, the difference is mainly due to the improvement of knowledge about malaria and the practice of preventive measures. Despite a lack of evidence of an actual improvement of the perception and attitude scores, these scores are higher than the knowledge and practice scores, both in participants and non-participants. Moreover, the evaluation of the intervention shows good use of the self-test and treatment of the kit (71%) [14]. Thus, as mentioned earlier, there may be a spillover effect of Malakit through the dissemination of information delivered during the training sessions by participants to other people working in gold mines [12, 14, 16]. A more accurate assessment of the level of exposure to the information delivered in Malakit would be needed to validate this hypothesis and estimate this effect.

### Towards malaria elimination: sustaining efforts and engaging communities

This study and evidence in the literature highlight the importance of remaining vigilant in the context of declining malaria incidence [26, 47–51]. Risk perception regarding malaria and behaviour changes play a significant role in the control and elimination of malaria since the only reservoir of the disease is human. Therefore, it is essential to raise and maintain awareness and a sustained level of information on malaria in the communities but also among health professionals working with communities [20, 21, 45, 46, 52, 53]. Mobilizing communities to eliminate malaria requires an understanding of their perceptions of health and disease and working with them to develop innovative educational programmes adapted to the sociocultural context [54, 55]. Educational tools of Malakit, such as the videos, were designed and tested with the gold miners, were appreciated by the participants and seem to be effective on behaviour changes [12, 15, 16], even if there is still room for improvement. Some countries have developed educational programmes using traditional arts such as music, theatre, storytelling or movies, to engage hard-to-reach communities through the health care system [56–59]. In this context, songs and music videos may be effective ways to reach out to gold miners, a highly mobile population. Participatory approach and future studies will help developing new tools to engage gold miners in malaria elimination.

However, the community-based approach requires investment in time, financial and human resources. Malaria must therefore remain a priority for countries – even in a context of low transmission— if the aim is to eliminate malaria [47, 60].

## Conclusions

Raising awareness and maintaining communities’ participation in malaria programmes is a major challenge given the observed decrease in risk perception and knowledge about malaria associated with the decrease in malaria transmission. The community-based and participatory approach of Malakit has proven to be effective in improving overall perceptions, knowledge, attitudes and practices related to diagnosis, treatment, and prevention of malaria among clandestine gold miners. The results of this study will be useful in modifying and improving the training of the participants before their receiving a kit. The integration of the Malakit strategy into malaria control programmes could help to improve the KAP of targeted communities and its transfer could be considered to other regions with residual malaria in remote areas. Innovative approaches and new tools have to be thought and developed with the communities to maintain their engagement in malaria elimination.

## Supplementary Information

**Additional file 1:** Information, education and communication tools and content

**Additional file 2:** Participants training tools

**Additional file 3:** Post-survey questionnaire

**Additional file 4:** Details of scores calculation

**Additional file 5:** KAP before (N=420) and after (N=381) Malakit intervention (%)

**Additional file 6:** KAP among included (N=247) and not included (N=107) participants in Malakit (%)

**Additional file 7:** Sensitivity analysis

## Data Availability

The datasets used and analysed during the current study are available from the corresponding author on reasonable request.

## Abbreviations

ACT: Artemisinin-based combination therapy
API: Annual parasite index
ATE: Average treatment effect
ATT: Average treatment effect on the treated
FG: French Guiana
CI: Confidence interval
IPTW: Inverse probability of treatment weighting
KAP: Knowledge, attitude, practice
OR: Odds ratio
PAHO: Pan American Health Organization
PS: Propensity score
PSM: Propensity score matching
SMD: Standardized mean difference
SDG: Sustainable development goals
WHO: World Health Organization
