# Supplementary material for: Impact of Malakit intervention on perceptions, knowledge, attitudes, and practices related to malaria among workers in clandestine gold mines in French Guiana: results of multicentric cross-sectional surveys over time
Source: Malar J. 2022 Dec 28;21:397. doi: 10.1186/s12936-022-04391-4 (PMC9795716; doi:10.1186/s12936-022-04391-4)
Supplement: Supplementary file 1 — Additional file 1: Information, education and communication tools and content [file 12936_2022_4391_MOESM1_ESM.docx]

## Supplementary material I. Information, education and communication tools and content

| Tool | Medium | Objectives | Content of messages |
| --- | --- | --- | --- |
| Illustrated posters | Displayed and explained by the facilitators during training | Support tool for the training dispensed by facilitators to impart knowledge about malaria and its treatment and introduce the explanation about when and how to use correctly the kit | - Poster “One infection – one appropriate treatment”: the importance of knowing the cause of the infection. The treatment of the kit will not work if the cause of the symptoms is not malaria. - Poster of parasites: If the treatment is not complete, the strongest parasites will remain, and with time the treatment will lose its effect on malaria. - Poster "Malaria parasite": the existence of P. vivax and P. falciparum. The RDT of the kit detects both. The treatment of the kit eliminates the parasite circulating in the body for both types and therefore, eliminates symptoms. However, if symptoms reappear one month later, it can be P. vivax which was dormant in the liver and is now waking up. In this case, it is important to consult a health professional.  - Poster of risky situations (include severe signs and the presence of a heart condition): Beware, in your case, malaria can be severe. Start the treatment and go to a health centre as soon as possible. |
| Information video | Mobile application | Presentation of the disease | Explanations on: vector, parasite, transmission, symptoms, diagnostic methods. |
| Prevention video | Mobile application | Presentation of the prevention measures against malaria | The risk of mosquito bites is higher during the night Use of mosquito nets  Use of repellent on the skin Advice to avoid places where mosquitoes can breed (avoid standing water, keep inside and outside clean |
| Animated video | -Displayed by the facilitators during the training -Shared with participants owning a smartphone, for further sharing on social networks Included in the Malakit app distributed to participants | Presentation of the malakit (object) and general presentation of the project | - Kit to detect and treat malaria in case of symptoms - Content of the kit: "3 RDTs and full malaria medication" - Importance of taking the entire malaria medication and risk of resistance to ACT - Kit given for free - Location of distribution sites |
| Illustrations and text about the RDT | Printed on the plastic RDT holder of the malakit  Displayed by the facilitators during the training | Instructions for performing a  self-RDT | *- "When you feel sick, the best is to visit a health centre" - "Be careful when the test is negative, do not use Coartem®" - "Take paracetamol if you feel fever or pain"* - Visual explanations on how to interpret the result (two lines = positive, one line in front of C = negative etc.)  - *“In case of positive RDT, take the treatment against malaria” - “In case of negative RDT, do not take the treatment against malaria” - “In case of invalid RDT, redo the test”* |
| Illustrations and text about the treatment | Printed on the plastic Treatment holder of the malakit  Displayed by the facilitators during the training | Explanations and instructions on when and how to take the different drugs of the kit | *- "If you do not feel better, try to consult a health centre" - "Be careful when the test is negative, do not use Coartem®"* - Paracetamol is to be taken in case of fever or pain (1000 mg, 3 times a day maximum) *- "If the result of the malaria test is positive, do the entire treatment"* - Primaquine is not allowed for pregnant women - *"Take 2 pills of primaquine with the Coartem®"* - Coartem®: "Take 4 pills in the morning and 4 pills in the evening during 3 days" - Importance of taking the entire malaria medication to eliminate the parasite, even if the fever disappears and you feel better. |
| RDT video | -Displayed by the facilitators during the training - Shared with participants owning a smartphone - Included in the Malakit app distributed to participants | Instructions for performing a  self-RDT | - Explanations on how to interpret the result  - In case of invalid RDT, redo the test - If the test line is clear and indefinite, consider the test as positive - The RDT waste should be disposed of in the yellow bag and given to the facilitators |
| Interactive module | Mobile application | Personal tutorial for malakit use in case of symptoms | - "*You should consult a medical doctor in a health centre*" - "*The malakit is not suitable for people under 15 years old*" - List of malaria symptoms (with pictograms) - Visual explanations on how to interpret the result of the RDT - List of risky situations which includes signs of severe malaria and a history of heart condition (with pictograms) - In case of signs of severe malaria: "*Beware*, *in your case, malaria can be severe. Start the treatment and go to a health centre as soon as possible*." - "*Beware, if you are pregnant or breastfeeding, do not take primaquine. Go to a health centre as soon as possible.*" - Treatment instructions - Reminder alerts for each intake of Coartem®, and a question after three days to confirm whether the medication was taken entirely - "*Sleep under a mosquito net to avoid malaria transmission.*" - "*You should consult a health centre for a medical opinion*" if the person does not feel better after the end of treatment.  - In case of a negative test: “*It is not recommended to take the treatment against malaria without positive test. You can take paracetamol in case of fever. If symptoms persist, do another test and consult a health centre*”. |
