# Supplementary figures and images for: Impact of Malakit intervention on perceptions, knowledge, attitudes, and practices related to malaria among workers in clandestine gold mines in French Guiana: results of multicentric cross-sectional surveys over time
Source: Malar J. 2022 Dec 28;21:397. doi: 10.1186/s12936-022-04391-4 (PMC9795716; doi:10.1186/s12936-022-04391-4)

## Supplementary material II. Participants training tools


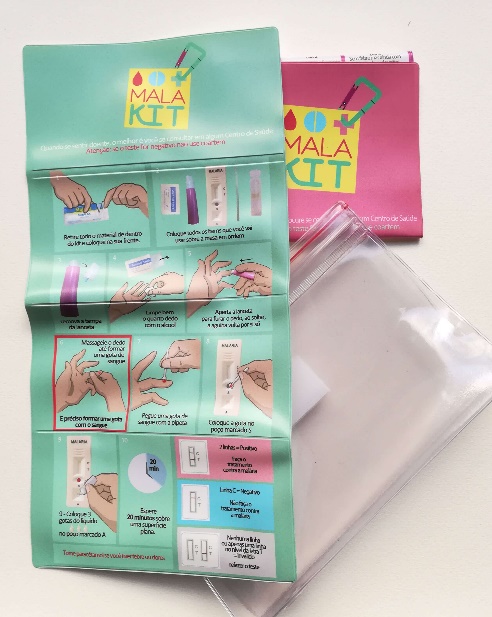

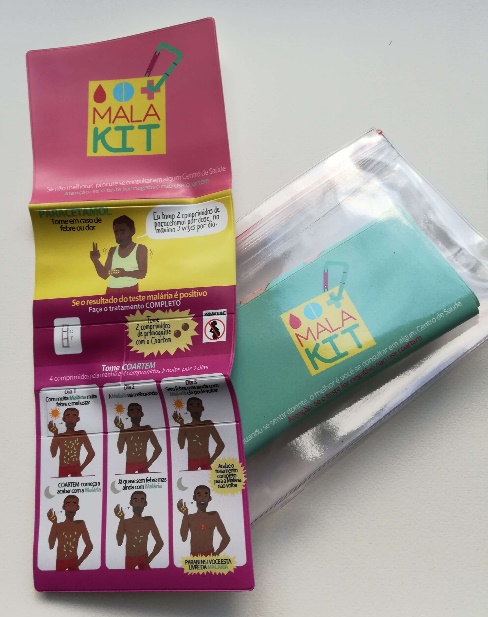

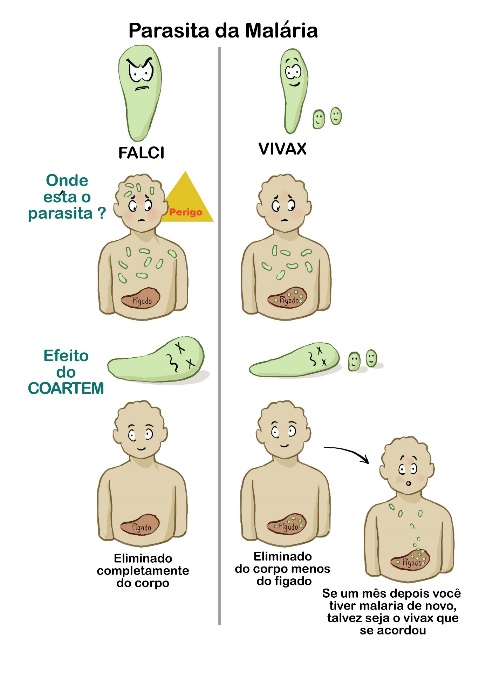

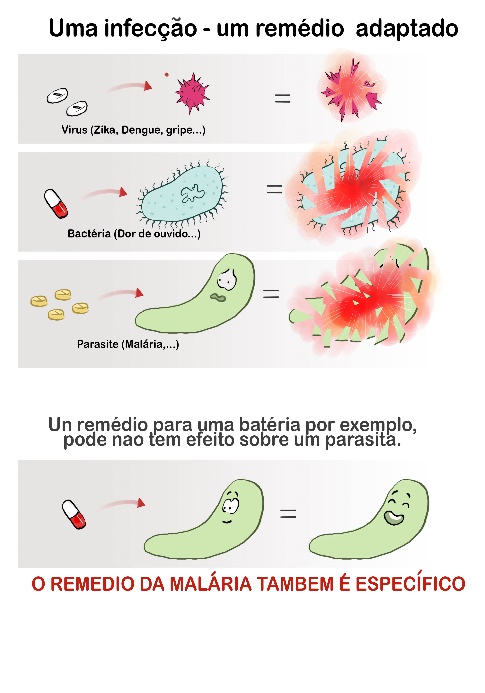

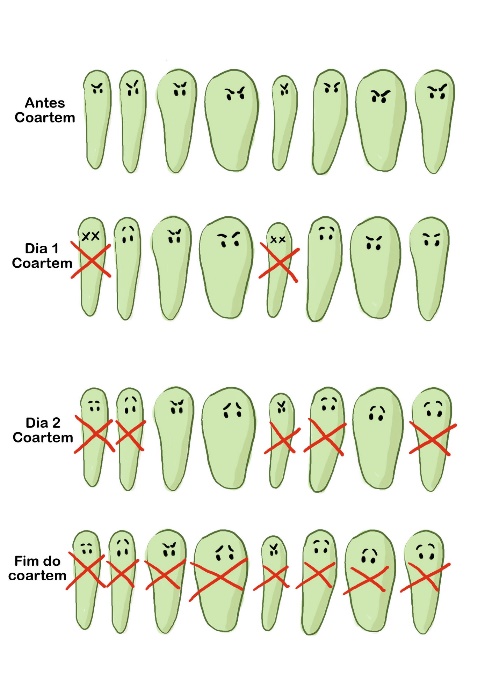

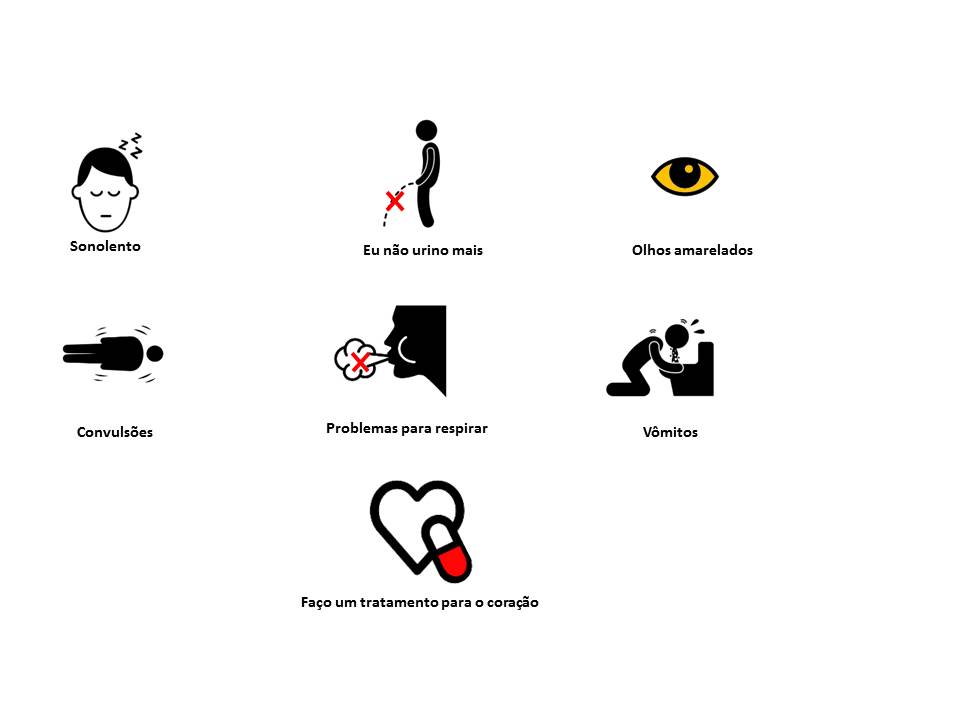

Supplement: Supplementary file 2 — Additional file 2: Participants training tools [file 12936_2022_4391_MOESM2_ESM.docx]
