# Supplementary material for: Impact of Malakit intervention on perceptions, knowledge, attitudes, and practices related to malaria among workers in clandestine gold mines in French Guiana: results of multicentric cross-sectional surveys over time
Source: Malar J. 2022 Dec 28;21:397. doi: 10.1186/s12936-022-04391-4 (PMC9795716; doi:10.1186/s12936-022-04391-4)
Supplement: Supplementary file 3 — Additional file 3: Post-survey questionnaire [file 12936_2022_4391_MOESM3_ESM.docx]

**Socio-demographic data**

**Q4 :** Gender : : 1. female  2. Male  3. Other: ...

**Q4-1: If female, pregnant** ?

0. no

1. yes: term: .............................

**Q5: Age** (years): ................

**Q6: In which country were you born?**

1. Brazil

2. French Guiana

3. Suriname

4. Guyana

5. Dominican Republic

6. Other: ......................

**Q6-1: If Brazil, specify state:**

1. Amapa

2. Para

3. Roraima

4. Maranho

5. Other: ............................

**Q7: What is your primary language?**

1. Portuguese

2. French

3. Dutch

4. English

5. Sranan-tongo

6. Other: ..........

**Q7-1: Do you speak Portuguese?**

1. commonly

2. A little

3. No

**Q8: What is your highest level of education?**

0. none

1. Primary

2. Secondary

3. Superior

4. Dnk

**Q9: Do you have social security in France?**

0. general social security

1. CMU

2. AME

3. No

4. Dnk

**Data related to gold panning activity**

**Q10: How long have you been working in gold panning**?

....................... days .............. ....... months / .................... years

**Q11: What site are you currently working at**?

......................................................................................................

**Q12: Do you know the name of the river and/or the village closest to the gold panning site where you are currently working?**

1. yes: river name: ........................................ Village name: .............................

2. No

3. Don't know

4. Do not wish to answer

**Q13: How long have you been at this (the last) gold panning site?**

............................days / .................................months /..............................years

**Q14: How far from here is the site? (In time)**

**Q15: How do you get to your current site? (Multiple answers possible)**

1. On foot

2. By quad bike

3. By canoe

4. by plane

5. Other: ....................................

6. Dnk

**Q16: What type of mine are you currently working on? What is the exploration method used?**

1. Alluvial

2. Well

3. Both

4. Other: .................................

**Q17: What are you doing on the site right now? "You can give two answers starting with the main activity". ( 1 2**

1. orpailleur

2. Machine operator

3. Sex worker

4. Transporter piroguier canoeiro

5. Bar tender

6. Store clerk

7. Store owner

8. Housekeeper/Cook

9. Street vendor  10. Porter

11. Unemployed

12. Other

13. Dnk

**Q18: Do you work:**  1. By day only

2. By night only

3. Both

4. Dnk

**Q19: How many people live in the same camp as you?**

1. <10

2. 11-50

3. 51-100

4. >100

5. Dnk

**Q20: Can you estimate the number of people at your workplace/camp who have not been out of the woods for more than 3 years?**

1. yes: .........................persons

2. No/dk

**Q21: In the past year, how many times have you left the forest for more than 3 days?**

**Q21-1: When was the last time you were out of the woods before this one?**

1. .................... days ago

2. ..................... months ago

3. ..................... years ago

4. Don't know

5. Do not wish to answer

**Q22: For what reason(s) are you leaving the mine?**  1. rest

2. Visit of the family

3. Medical care

4. Procurement

5. Gendarmerie/armyraid

6. Other: ....................................

**Q23: Do you get out of the forest especially at certain times of the year?**  1. yes, period: ...............................(check the months of the year mentioned)

2. No

3. Dnk

**Q24: In which major cities did you visit this year?**  1. oiapoque

2. Macapa

3. Belem

4. Sao Luis

5. Fortaleza

6. Manaus

7. Cayenne

8. Kourou

9. Saint Laurent

10. Albina

11. Paramaribo

12. Other: .................................

13.other: .................................

**Q25: Where did you work before the current site?**  1. Gold panning site in Guyana: name: ................

2. Gold panning site in Brazil

3. Gold panning site in Suriname

4. Other: .............

**Q26: On how many different mines have you worked in the last 3 years**  1. in Guyana: ...................... times

2. In Brazil: ..........................fois

3. In Suriname: ................................ times  4. Other : In ...................................fois

**Q27: What are the top three health issues you encounter at sites**

**1 2 3**

1. Malaria

2. Cutaneous leishmaniasis

3. Digestive disorders

4. Musculoskeletal disorders

5. Headaches    6. "flu

7. HIV

8. Skin problems other than leishmaniasis (macaque worms, mycosis, etc...)

9. Other......................

10. Other ......................

11. Other......................

**Knowledge, attitudes and practices towards malaria**

**Q28: What do you think causes malaria?**

**1 2 3**

1. mosquito (without specifying the bite): **"can you specify?"**

2. Mosquito bite

3. ingestion of unsafe river water

4. Living next to a dirty river

5. Living in a dirty environment

6. Other: ..............................................................................

7. Other: ..............................................................................

8. Other: ..............................................................................

9. Dnk

**Q29: Can you indicate whether the following statements are true or false:**

|  | **Fake** | **True** | **nsp** |
| --- | --- | --- | --- |
| 1. Malaria treatment can be stopped when you feel better | 0 | 1 | 2 |
| 1. Malaria kills people | 0 | 1 | 2 |
| 1. Drugs from health centers/hospitals are better than those purchased on the black market | 0 | 1 | 2 |
| 1. Malaria can be cured on its own without drugs | 0 | 1 | 2 |
| 1. You should be tested for malaria before taking any malaria treatment | 0 | 1 | 2 |
| 1. After a negative malaria test, it's best to take malaria treatment anyway, just to be sure | 0 | 1 | 2 |

**Q30: What do you think of Artecom? O que voce pensa do artecom? (Several answers possible)**

1. I do not know

2. It is a good drug  3. it doesn't work well

4. It is too expensive

5. it has too many side effects

6. This treatment is better than that of the health centers  7. Other..................................

8. Dnk

**Q31: What are the symptoms of malaria?**

1. fever

2. Vomiting

3. Headaches

4. Abdominal pain

5. Curves

6. Thrills

7. Feeling of cold

8. Other: ..........................................

9. Other: ............................................

10. Other: ..............................................

**Q32: Do you protect yourself against mosquitoes?**

1. always yes

2. Yes, often

3. Yes, sometimes

4. Never

5. Dnk

**Q32-1: If so, how? Se "Sim", como? (Several answers possible)**

1. mosquito net

2. Repellents

3. Long clothes

4. Prophylactic drugs

5. Stay away from sewage

6. Do not drink dirty water

7. Other: ..........................................

8. Dnk

**Q33: Did you sleep under a mosquito net during your last night at the site?**

0. no Não (>

1. yes Sim (>

2. Nsp Não sei

**Q33-1: If yes: was the net impregnated?**  0. no

1. yes  2. Other

3. Dnk

**Q33-2: If not, why not**?  1. uncomfortable

2. No disease on the site

3. Does not see the point

4. Too expensive

5. Too restrictive

6. Makes it difficult to escape quickly if needed

7. Has been burned / destroyed

8. Has been given

9. Has been loaned

10. Don't have any :...............................

11. Other: ..................

**Q34: Have you ever had malaria?**

0. never/

1. yes ....... times

5. Don't know/
