# Supplementary material for: Impact of Malakit intervention on perceptions, knowledge, attitudes, and practices related to malaria among workers in clandestine gold mines in French Guiana: results of multicentric cross-sectional surveys over time
Source: Malar J. 2022 Dec 28;21:397. doi: 10.1186/s12936-022-04391-4 (PMC9795716; doi:10.1186/s12936-022-04391-4)
Supplement: Supplementary file 4 — Additional file 4: Details of scores calculation [file 12936_2022_4391_MOESM4_ESM.docx]

## Supplementary material IV: Details of scores calculation

| **ITEM** | **POINTS** | **PRE-INTERVENTION SURVEY** | **POST-INTERVENTION SURVEY** |
| --- | --- | --- | --- |
| **PERCEPTION SCORE** | | | |
| What are the three main health problems you encounter on the sites? | 1 if malaria cited as one of the three main health problems, 0 otherwise | X | X |
| Malaria kills people. | 1 if “yes”, 0 otherwise | X | X |
| Malaria can be cured on its own without drugs. | 1 if “yes”, 0 otherwise | X | X |
| **KNOWLEDGE SCORE** | | | |
| What do you think causes malaria?” | 1 if “mosquito” or “mosquito bite”, 0 otherwise | X | X |
|  | 2 if “mosquito bite”, 1 if “mosquito”, 0 otherwise |  | X |
| What are the symptoms of malaria? | 1 if at least 3 symptoms, 0 otherwise | X | X |
| **ATTITUDE SCORE** | | | |
| You can stop treatment for malaria when you feel better. | 1 if “no”, 0 otherwise | X | X |
| A malaria test should be done before taking malaria treatment. | 1 if “yes”, 0 otherwise |  | X |
| After a negative malaria test, it is better to take malaria treatment anyway, just to be sure. | 1 if “no”, 0 otherwise | X | X |
| Medicines from health centres/hospitals are better than those bought on the black market. | 1 if “yes”, 0 |  | X |
| **PRACTICE SCORE** | | | |
| Do you protect yourself against mosquitoes? | 1 if “Yes, always” or “Yes, often”, 0 otherwise | X | X |
| Did you sleep under a mosquito net during your last night on site? | 1 if “Yes”, 0 otherwise | X | X |
| **TOTAL KAP SCORE** | | | |
| KAP 9 | Sum of each item | X | X |
| KAP 12 | Sum of each item |  | X |
