# Supplementary material for: Impact of Malakit intervention on perceptions, knowledge, attitudes, and practices related to malaria among workers in clandestine gold mines in French Guiana: results of multicentric cross-sectional surveys over time
Source: Malar J. 2022 Dec 28;21:397. doi: 10.1186/s12936-022-04391-4 (PMC9795716; doi:10.1186/s12936-022-04391-4)
Supplement: Supplementary file 5 — Additional file 5: KAP before (N=420) and after (N=381) Malakit intervention (%) [file 12936_2022_4391_MOESM5_ESM.docx]

## Supplementary material V: KAP before (N=420) and after (N=381) Malakit intervention (%)
