# Supplementary material for: Impact of Malakit intervention on perceptions, knowledge, attitudes, and practices related to malaria among workers in clandestine gold mines in French Guiana: results of multicentric cross-sectional surveys over time
Source: Malar J. 2022 Dec 28;21:397. doi: 10.1186/s12936-022-04391-4 (PMC9795716; doi:10.1186/s12936-022-04391-4)
Supplement: Supplementary file 7 — Additional file 7: Sensitivity analysis [file 12936_2022_4391_MOESM7_ESM.docx]

## Supplementary material VII: Sensitivity analysis

Table 1. KAP 12 score PSM sensitivity analysis using Rosenbaum Sensitivity Test for Wilcoxon signed rank p.value

| Γ p lower bound p upper bound |
| --- |
| 1.0 9e-04 0.0009 |
| 1.1 2e-04 0.0033 |
| 1.2 1e-04 0.0090 |
| 1.3 0e+00 0.0204 |
| 1.4 0e+00 0.0399 |
| 1.5 0e+00 0.0693 |
| 1.6 0e+00 0.1094 |

Gamma (Γ ) is the sensitivity parameter. It is an odds ratio that measures the degree of departure from random assignment of treatment (in randomised studies, Γ =1). Two subjects with the same observed characteristics may differ in the odds of being treated by at most a factor of Γ.

Here, p>0.05 for Γ = 1.4: the odds ratio would have to change by a factor of 1.4 to render the estimates of the ATT of Malakit on KAP 12 statistically insignificant at a significancy level of 5%. That means that for two subjects identical on matched covariates, one might be 1.4 times as likely as the other to be included in Malakit because they differ in terms of an unobserved covariate (hidden bias).

Table 2. KAP 12 score after IPTW sensitivity analysis using optimal trimming among participants and non-participants in Malakit

| **Score** | **Not included in Malakit (N=245)**  mean (sd) | **Included in Malakit**  **(N=107)**  mean (sd) | **Coefficient [IC95%]** | **p.value** |
| --- | --- | --- | --- | --- |
| KAP 12 | 8.62 (1.52) | 9.32 (1.53) | 0.70 [0.34 ; 1.05] | <0.001* |

After optimal trimming, two participants were removed from the non-participant’s group. No difference was observed with the IPTW analysis in the KAP 12 score after optimal trimming compared to the IPTW analysis without trimming.
